# Editorial: Advances in the Diagnosis and Control of Johne's Disease

**DOI:** 10.3389/fvets.2021.771891

**Published:** 2021-10-04

**Authors:** Marta Alonso-Hearn, Miguel Salgado, Kumudika de Silva

**Affiliations:** ^1^NEIKER- Basque Institute for Agricultural Research and Development, Department of Animal Health, Basque Research and Technology Alliance (BRTA), Derio, Spain; ^2^Facultad de Ciencias Veterinarias, Instituto de Medicina Preventiva Veterinaria, Universidad Austral de Chile, Valdivia, Chile; ^3^Faculty of Science, School of Veterinary Science, The University of Sydney, Sydney, NSW, Australia

**Keywords:** Johne's disease, diagnosis, control, vaccines, genetic resistance

We are delighted to have contributed as Guest Editors to this special Research Topic entitled: “Advances in the Diagnosis and Control of Johne's Disease.” This Research Topic is a collection of 15 manuscripts (14 original research papers and 1 minireview article) from 112 authors. *Mycobacterium avium* subsp. *paratuberculosis* (MAP) causes Johne's disease or paratuberculosis (PTB), an infectious chronic enteritis in domestic and wildlife ruminants with important welfare, economic, and potential public health consequences. This Research Topic explores novel technologies that have the potential both to advance our understanding of the immunopathology of MAP infection and to improve current diagnostic and control strategies.

The contribution of host genetic factors is one of the fundamental issues in understanding PTB pathogenesis and host resistance, tolerance, and resistance. Microarrays and high-throughput next-generation sequencing (NGS) are the cutting-edge technologies for genomics and transcriptomics studies. In this special issue, two studies by Kiser et al. and Okuni et al. analyzed loci associated with disease susceptibility that could be used by producers to select cattle that were less susceptible to PTB. Kiser et al. identified 18 single-nucleotide polymorphisms (SNPs) associated with MAP tissue infection through resequencing and fine-mapping of the region between the *EDN2* and *HIVEP3* genes in Holstein and Jersey breeds. Okuni et al. surveyed a population of Ankole cattle in Uganda, where there are no control measures against PTB, to determine associations between ELISA test results and allelic variants in the *SLC11A1, IFN*γ, *TLR4, NOD2*, and *PGLRYRP1* genes. NGS-based RNA sequencing (RNA-Seq) can identify and quantify a variety of RNA species including long non-coding RNAs (lncRNAs). lncRNAs play an important role in regulating the immune response of macrophages to bacterial infections, including PTB. The novelty of the study by Marete et al. is that it identified genome-wide lncRNAs with potential roles in host immunity using RNA-Seq from macrophages of Canadian Holsteins naturally infected with MAP. In parallel to the revolutionary progress of NGS technology, direct analysis in real-time coupled to high-resolution mass-spectrometry (DART-HRMS) is another innovative approach to overcome the lack of efficient diagnostic methods in the subclinical stages of PTB. This technology allowed the identification of several metabolic markers associated with infected and infectious stages of PTB as was demonstrated in this special issue by Tata et al.

Diagnostic tests are central to limit the spread of PTB and are hampered by the lack of MAP-specific targets. In this special Research Topic, several studies describe MAP-specific genes and antigens that have the potential to improve the specificity of PTB diagnostic tests. Bannantine et al. aimed to define a more complete catalog of *M. avium* subspecies-specific genes. They confirm 86 genes as MAP-specific, seven as *M. avium* subspecies *avium*-specific, and three as *M. avium* subspecies *hominissuis*-specific. A single-tube PCR reaction was conducted as a proof-of-concept to quickly distinguish *M. avium* complex (MAC) strains. Karuppusamy et al. evaluated the potential of ELISAs based on six antigenically distinct recombinant MAP cell envelope proteins (SdhA, FadE25_2, FadE3_2, Mkl, DesA2, and hypothetical protein MAP1233) to detect antibodies against MAP in the sera of infected cattle. Their results suggested that antigenically distinct MAP cell envelope proteins and antibodies to these proteins may have the potential to detect MAP infection in dairy cattle. Unlike other MAC members, MAP does not produce glycopeptidolipids on the surface of the cell wall but a lipopentapeptide called L5P. Bay et al. produced by chemical synthesis a water-soluble variant of L5P and evaluated this compound for the serological diagnosis of MAP using well-defined serum banks. L5P and its water-soluble derivative used alone in ELISA had lower sensitivity (Se 82% for L5P and Se 62% for the water-soluble variant of L5P) compared to the Se of a commercial test (98%). The fact that L5P could not be validated in the context of ovine PTB highlighted the need to better characterize the antigens expressed from the different genetic lineages of MAP. In this context, Mizzi et al. investigated sheep MAP isolates from Australia and New Zealand using whole-genome sequencing. The genetic differences identified in this report may represent important epidemiological and virulence traits specific to sheep MAP strains. This knowledge could potentially contribute to improved vaccine development and control measures for these strains.

It is also widely acknowledged that the current tests for the diagnosis of PTB do not detect all MAP-infected animals. Consequently, PTB control efforts based on fecal culture and serum- or milk-ELISA results have not been as effective as national governments would have liked. In this special issue, several methods to improve MAP detection were described. Vitense et al. described a method to improve detection of MAP from fecal and tissue samples measuring volatile organic compounds. Biomarkers to improve differentiation between PTB disease outcomes have been studied in recent years. The use of the alpha 2 macroglobulin as a biomarker for PTB is presented by Park et al. where they assessed serum proteins in MAP exposed animals. Interferon-gamma (IFNγ) release from antigen-stimulated whole blood is a well-known marker of MAP exposure but is currently not used as a diagnostic test. Corneli et al. used antigens (Johnins) prepared from MAP isolates from a specific region and monitored herds over a few years. This allowed them to propose new criteria to better interpret IFN-γ test results. Hatate et al. described a new electrochemical detection method for MAP antibody testing that could distinguish samples of MAP-infected cattle from those of uninfected cattle with greater separation enabling on-site diagnostic testing for PTB.

Phage-based methods are a relatively recent addition to the PTB diagnostic toolbox. In this special issue, Grant reviewed the basis of the phage-based tests and described modifications made to the original plaque assay-based phage amplification assay (FASTPlaqueTBTM) over time to simplify the assay and make it more user-friendly. Finally, the performance of the phage assays for testing veterinary specimens (bovine milk, blood, and feces) relative to current PTB diagnostic methods (culture, fecal PCR, and blood-ELISA) was summarized. In addition, Kubala et al. developed a method of blood preparation for phage assays. Their results showed that this improved sample preparation method and Actiphage blood testing can be used to test blood samples from deer, and the full diagnostic potential of the method was evaluated.

Rasmussen et al. delve into modeling the effectiveness and benefits of long-term strategies for controlling PTB in dairy herds, focusing on the Canadian context. Current vaccines only induce protective immunity or suppress MAP shedding in some animals. Importantly, these factors were considered in this analysis. While the economic viability of PTB control strategies is the central theme, this work also holds clues for future PTB vaccine developers when testing novel vaccines for their ability to control the spread of disease.

We hope you enjoy reading and reflecting on these important topics while developing new integrated and multidisciplinary collaborations which will enable better detection of MAP infection and strategies for controlling Johne's disease.

## Author Contributions

All authors listed have made a substantial, direct and intellectual contribution to the work, and approved it for publication.

## Conflict of Interest

The authors declare that the research was conducted in the absence of any commercial or financial relationships that could be construed as a potential conflict of interest.

## Publisher's Note

All claims expressed in this article are solely those of the authors and do not necessarily represent those of their affiliated organizations, or those of the publisher, the editors and the reviewers. Any product that may be evaluated in this article, or claim that may be made by its manufacturer, is not guaranteed or endorsed by the publisher.

